# 

**Published:** 1890-03-01

**Authors:** 


### THE HOSPITAL

A WEEKLY JOURNAL OF
Science, Medicine, Nursing and Philanthropy.

SATURDAY, MARCH 1st, 1890.

**Hypnotism Medically Considered** - 335
**Words of Consolation.**—Freedom - 336
**The Doctor's Pulpit.**—Food and Force - 337
**The "Diphtheria House" at Zurich** - 338
**Editor's Letter Box** - 338
**The Town Dweller** - 339
**March** - 340
**Everybody's Page** - 341
**Notes and News** - 342
**Annotations.**—Dressed Dolls—A Bacillary Check—Eminent Men's Wives— - 344
**Passing Topics.**—Ought Members of the Active Medical Staffs to sit upon Hospital Boards? - 350

**The Practitioner's Mirror and Retrospect:**
Medicine—Diseases of the Chest—Goitre—A New Symptom—Vertigo—Croup - 345
Surgery—Hæmorrhoids - 346
Therapeutics—Saccharin—Insects in Drugs—Alum Injection for Dysentery—The Ice Treatment—Novel Use of Mercury—The Use of Antimony - 346
Climatology—Winter Climate of the Nile - 347
Dietetics—Infants' Food - 347
New Drugs, Appliances, and Things Medical—The Lactotherme—The Electric Cystoscope - 348
**Earlswood As It Is** - 349
**Scraps and Gleanings** - 350

### EXTRA SUPPLEMENT—THE NURSING MIRROR.

**En Passant.**—Short Items—East London Nursing Society—North London Nursing Association—Thousands of Pounds for the National Pension Fund—The Leeds Institution—Naval Nursing Sisters—South Devon Hospital - lxxxix
**Nursing Lectures.** By Percy Lewis, M.D. IV.—The Care of the Chest - xc
**Everybody's Opinion.**—The Hospital Ship "Hamadryad," Cardiff Docks—Who will Help? - xci
**Examination Questions** - xci
**Presentation.—Death in Our Ranks** - xcii
**New Appliances.—Notes and Queries** - xcii
**Vacancies** - xcii
**Amusements and Relaxation** - xcii

